# Conversion of an amide to a high-energy thioester by *Staphylococcus aureus* sortase A is powered by variable binding affinity for calcium

**DOI:** 10.1038/s41598-018-34752-6

**Published:** 2018-11-06

**Authors:** Xiao Wang, Jia-Liang Chen, Gottfried Otting, Xun-Cheng Su

**Affiliations:** 1 State Key Laboratory of Elemento-Organic Chemistry, Department of Chemical Biology, College of Chemistry and Collaborative Innovation Center of Chemical Science and Engineering (Tianjin), Nankai University, Tianjin, 300071 China; 2 Research School of Chemistry, Australian National University, Canberra, ACT 2601 Australia

## Abstract

Thioesters are key intermediates in biology, which often are generated from less energy-rich amide precursors. *Staphylococcus aureus* sortase A (SrtA) is an enzyme widely used in biotechnology for peptide ligation. The reaction proceeds in two steps, where the first step involves the conversion of an amide bond of substrate peptide into a thioester intermediate with the enzyme. Here we show that the free energy required for this step is matched by an about 30-fold increase in binding affinity of a calcium ion at the calcium binding site of SrtA, which is remote from the thioester bond. The magnitude of this allosteric effect highlights the importance of calcium for the activity of SrtA. The increase in calcium binding affinity upon binding of substrate not only achieves catalytic formation of an energy-rich intermediate in the absence of nucleotide triphosphates or any tight non-covalent enzyme-substrate interactions, but is also accompanied by accumulation of the labile thioester intermediate, which makes it directly observable in nuclear magnetic resonance (NMR) spectra.

## Introduction

Thioesters play key roles in many biochemical and metabolic reactions. For example, peptide hydrolysis by cysteine proteases^1^, peptide bond rearrangements in inteins^2^, labeling of proteins with ubiquitin for subsequent degradation or other cellular functions^3^, and peptide-bond ligation by sortases^4^ all proceed via thioester intermediates. Beyond the formation of transient thioester bonds in enzymatic reactions, energy-rich thioester bonds are also of key importance in evolutionary ancient cofactors such as acetyl coenzyme A, leading to the hypothesis of a “thioester world” as an early precursor to life, prior to the emergence of nucleotide triphosphates as the general energy currency in biology^5^. While it is uncontroversial to think of thioester bonds as transient, energy-rich intermediates in an enzymatic reaction path, it is less obvious how an enzyme could generate a stable thioester by conversion of a relatively energy-poor amide bond in any appreciable yield without assistance from ATP-hydrolysis or harnessing of the free energy of binding between substrate and enzyme^6^.

**Figure 1:**
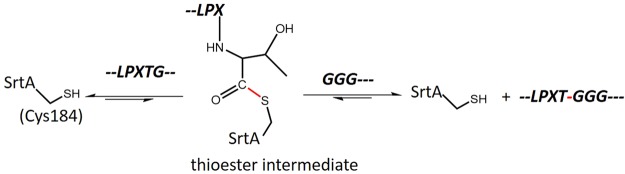
Reaction scheme of the transpeptidation process catalyzed by SrtA.***–****LPXTG–*and *GGG****–***denote peptides containing the canonical LPXTG and polyglycine motif, respectively.

Sortase A (SrtA) from *Staphylococcus aureus* anchors surface proteins to the bacterial cell wall, which makes it a key virulence factor. It is an enzyme conserved across all Gram-positive bacteria which also functions as a catalyst of biofilm formation, making SrtA a prime target of new antibiotic drugs^7–9^. Furthermore, due to its unique peptide ligation capabilities, SrtA is widely used as a ligase for protein modifications both *in vitro* and in cells as well as for peptide synthesis^10–16^. The reaction catalyzed by SrtA involves, as the first step, the conversion of a backbone amide of substrate peptide (namely the peptide bond between the C-terminal threonine and glycine residues in polypeptides containing the LPXTG motif, where X can be any amino acid) into a thioester intermediate (namely with the active-site cysteine residue, Cys184)^6,8,9^. In a second step, the energy of the thioester intermediate is used to form an amide bond between the LPXT peptide and a polyglycine peptide that serves as a second substrate (Fig. 1).

Calcium is known to enhance the enzymatic activity of SrtA^17,18^, but, as the calcium binding site is far from the active site of the enzyme^17–22^, its contribution is indirect. Comparison of the structure of apo-SrtA with the structure of a disulfide-bonded SrtA-substrate analogue determined in the presence of 20 mM CaCl_2_ indicated that the combined effect of substrate and calcium binding leads to a closed conformation of the enzyme, which involves the tying of a flexible polypeptide loop (the β6/β7 loop) to the core of the protein^22^. In the absence of substrate, calcium slows the dynamics of this loop^18^ and supports the closed conformation of the enzyme as indicated by computer simulations^23–25^. Kinetic data at pH 7.5 indicated that bound calcium enhances the substrate binding affinity about 4-fold, while the dissociation constant of calcium from SrtA was reported to be about 2.2 mM at pH 6.2^18^. In the present work, we conducted experiments with less salt present and measured an about 30-fold greater calcium binding affinity by isothermal calorimetry (ITC) and NMR at pH 6.4. More importantly, calcium was found to bind even more tightly in the presence of bound substrate peptide, and the labile thioester intermediate of SrtA is shown to accumulate sufficiently in the presence, but not in the absence, of calcium, allowing its direct observation by NMR spectroscopy. The increased concentration of the intermediate enhances the overall turnover rate of the enzyme.

Wild-type SrtA without calcium hardly binds substrates containing the canonical LPXTG motif. Similarly, the active-site mutant C184A, which maintains the structure of the wild-type protein, binds peptide substrate only very weakly irrespective of the presence of calcium. In contrast, we observed that a disulfide-bonded analogue of the thioester intermediate binds calcium with greatly enhanced affinity. While the calcium binding affinity of the native thioester intermediate could not be measured directly, it must likewise be greatly enhanced as the intermediate can be observed even with small amounts of calcium. This suggests that SrtA gains the free energy required to convert an amide bond into an energy-rich thioester bond from tighter coordination of a Ca^2+^ ion rather than from favorable non-covalent protein-substrate interactions. SrtA thus presents an unusual case of allosteric enzyme activation, where increased metal binding affinity allows the enzyme to perform an energetically unfavorable reaction. It is an efficient way to produce a reactive thioester intermediate on the outer surface of a Gram-positive bacterium and to regulate the enzyme activity in a calcium-dependent manner.

## Results

### Calcium is essential for the enzymatic reactivity of SrtA

**Figure 2:**
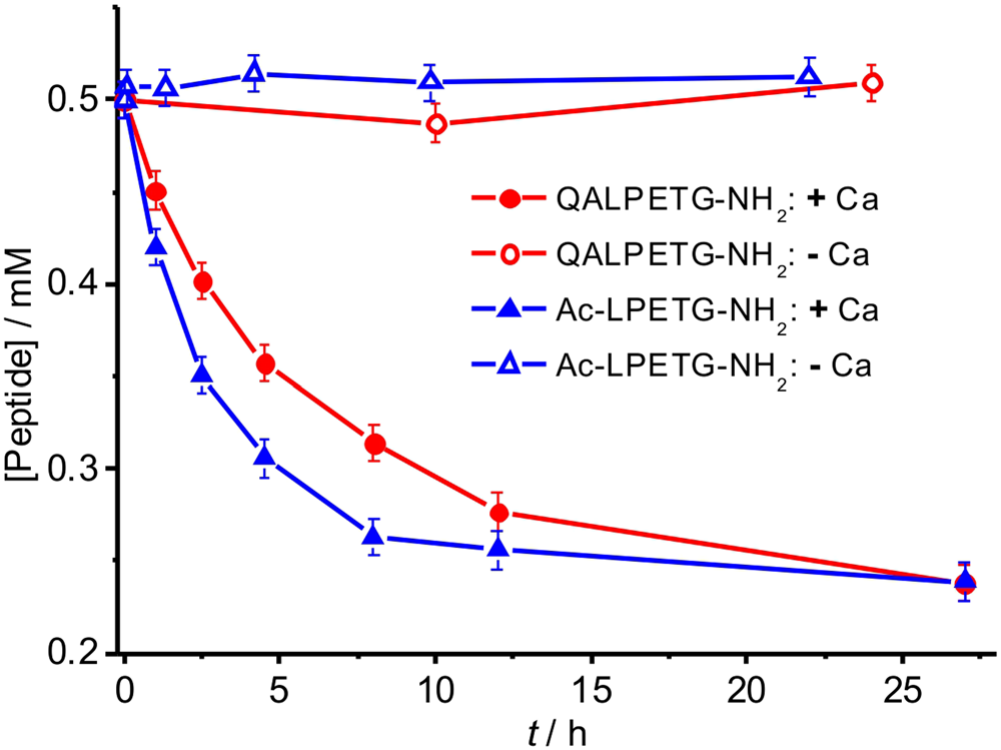
Calcium is a critical co-factor for the transpeptidation reaction of substrate peptide catalyzed by SrtA. Reaction monitored by the decrease of the ^1^H NMR signals of the C-terminal NH_2_ protons of the intact substrate peptides, for reaction mixtures of 0.01 mM SrtA in 20 mM Tris-HCl (pH 7.2) with 1.0 mM GGG and either 0.5 mM QALPETG-NH_2_ (red solid circle: with Ca^2+^; red open circle: without Ca^2+^) or Ac-LPETG-NH_2_ (blue solid triangle: with Ca^2+^; blue open triangle: without Ca^2+^). Samples without calcium contained 0.2 mM EDTA. Samples with calcium contained 0.1 mM CaCl_2_.

Five substrate peptides with different termini preceding and following the canonical LPXTG motif were synthesized (Fig. S1), and the catalysis of the transpeptidation and hydrolysis reactions of these substrates by SrtA was assessed in the presence and absence of calcium. The five substrate peptides displayed very different reactivities in the transpeptidation reaction in the presence of calcium, in that only the peptides containing amide bonds both preceding and following the LPXTG motif, Ac-LPETG-NH_2_ and QALPETG-NH_2_, displayed any significant reactivity. All other peptides remained unchanged with regard to transpeptidation and hydrolysis during incubation for 24 h. In line with a previous analysis^20^, these results indicate that charged termini cannot be tolerated, i.e. recognition of the canonical LPXTG motif alone is not sufficient for catalytic activity. In the presence of calcium, the transpeptidation reaction reached equilibrium in about 10 hours (Fig. 2). Hydrolysis of the thioester, which is an undesired side reaction, was much slower than transpeptidation (Fig. S2). Ac-LPETG-NH_2_ was slightly more reactive than QALPETG-NH_2_.

To assess the importance of calcium, we repeated the experiments in the absence of calcium. Any spurious traces of calcium were removed by the addition of 0.2 mM EDTA (EDTA does not interact with SrtA, as indicated by unchanged chemical shifts in ^15^N-HSQC spectra). In this case, there was no evidence for transpeptidation activity with any of the peptide substrates and the NMR signals of the peptides did not change noticeably during incubation (Fig. 2). In contrast to previous experiments, which reported significant residual catalysis by calcium-free SrtA^17^, our results point to a more fundamental role of calcium in SrtA catalysis.

### SrtA binds substrate peptides very weakly

**Figure 3:**
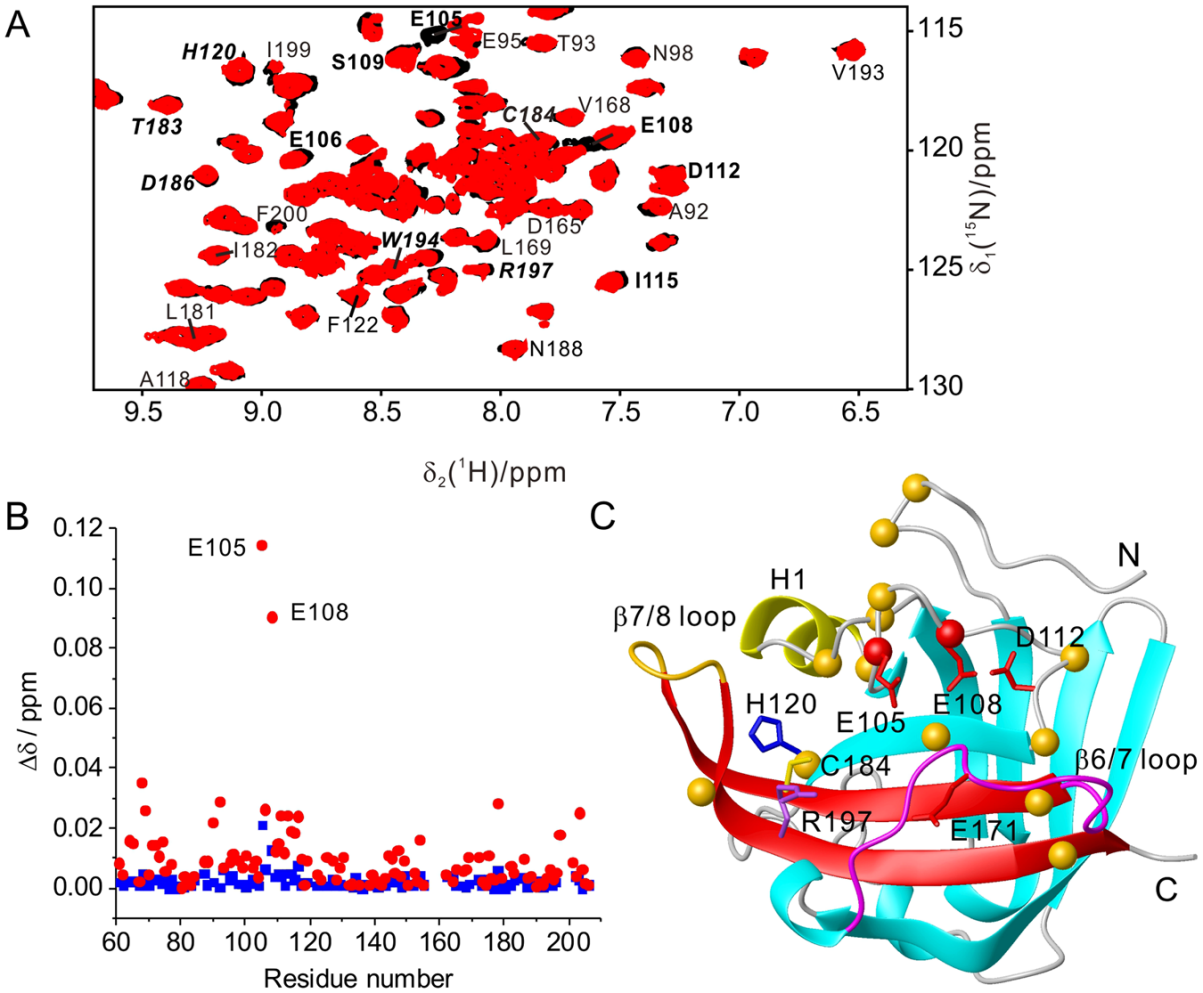
Titration of SrtA with substrate perturbs the calcium binding site more strongly than the active site. (**A**) Superimposition of ^15^N-HSQC spectra recorded for 0.10 mM SrtA in the absence (black) and presence (red) of 2.0 mM Ac-LPETG-NH_2_ in 20 mM MES, pH 6.4. Cross-peaks of residues close to the calcium binding motif are labeled in bold. Residues lining the active site are labeled in italics. (**B**) Chemical shift perturbations of 0.1 mM SrtA caused by the addition of 2.0 mM substrate peptide QALPETG-NH_2_ (squares) or Ac-LPETG-NH_2_ (circles). Chemical shift perturbations were calculated as Δδ = Sqrt[(Δδ_H_)^2^ + (Δδ_N_/10)^2^], where Δδ_H_ and Δδ_N_ are the backbone amide chemical shift differences in the ^1^H and ^15^N dimension, respectively. (**C**) Map of the chemical shift differences in (B) on the SrtA structure (PDB code: 1T2P) due to addition of Ac-LPETG-NH_2_. Backbone C^α^ atoms of residues with significant amide chemical shift changes are highlighted in red (Δδ  ≥ 0.04 ppm) and yellow (0.02 ppm ≤ Δδ  < 0.04 ppm).

If the transpeptidation reaction of SrtA depends on the presence of calcium, does calcium merely assist in substrate binding or does it participate in the chemical reaction? To answer this question, we analyzed the binding of substrate peptides to SrtA by high-resolution NMR spectroscopy. Without calcium, even a 20-fold excess of substrate peptide caused only very small chemical shift perturbations (CSP) in the ^15^N-HSQC spectrum of SrtA (Figs 3 and S3). Mapping the CSPs on the protein structure identified the largest changes for amino acids near the calcium binding motif (Fig. 3), whereas residues near the active site (defined by His120, Cys184, and Arg197) and those in the flexible β6/β7 and β7/β8 loops were less affected. In general, SrtA displayed larger CSPs with Ac-LPETG-NH_2_ than QALPETG-NH_2_ (Fig. 3). In either case, the binding affinities were too weak for quantitative determination of the binding constant by NMR titration.

To characterize the interaction of SrtA and substrate peptide in the presence of calcium, we studied peptide binding to the active-site mutant C184A. The mutation did not affect the overall structure of the protein, as large CSPs were observed only for a few residues close to C184 (Fig. S4). In addition, conservation of the 3D structure was confirmed by a 3D NOESY-^15^N-HSQC spectrum, which showed fully conserved cross-peak patterns (data not shown). Wild-type SrtA and the C184A mutant shared a similar binding affinity for calcium with dissociation constants, *K*_d_, of 70 and 90 μM, respectively, determined for a 1:1 binding model by NMR (Fig. S5). A previous NMR analysis reported much weaker binding (*K*_d_ ~2 mM) but was conducted in the presence of salt^18^.

**Figure 4:**
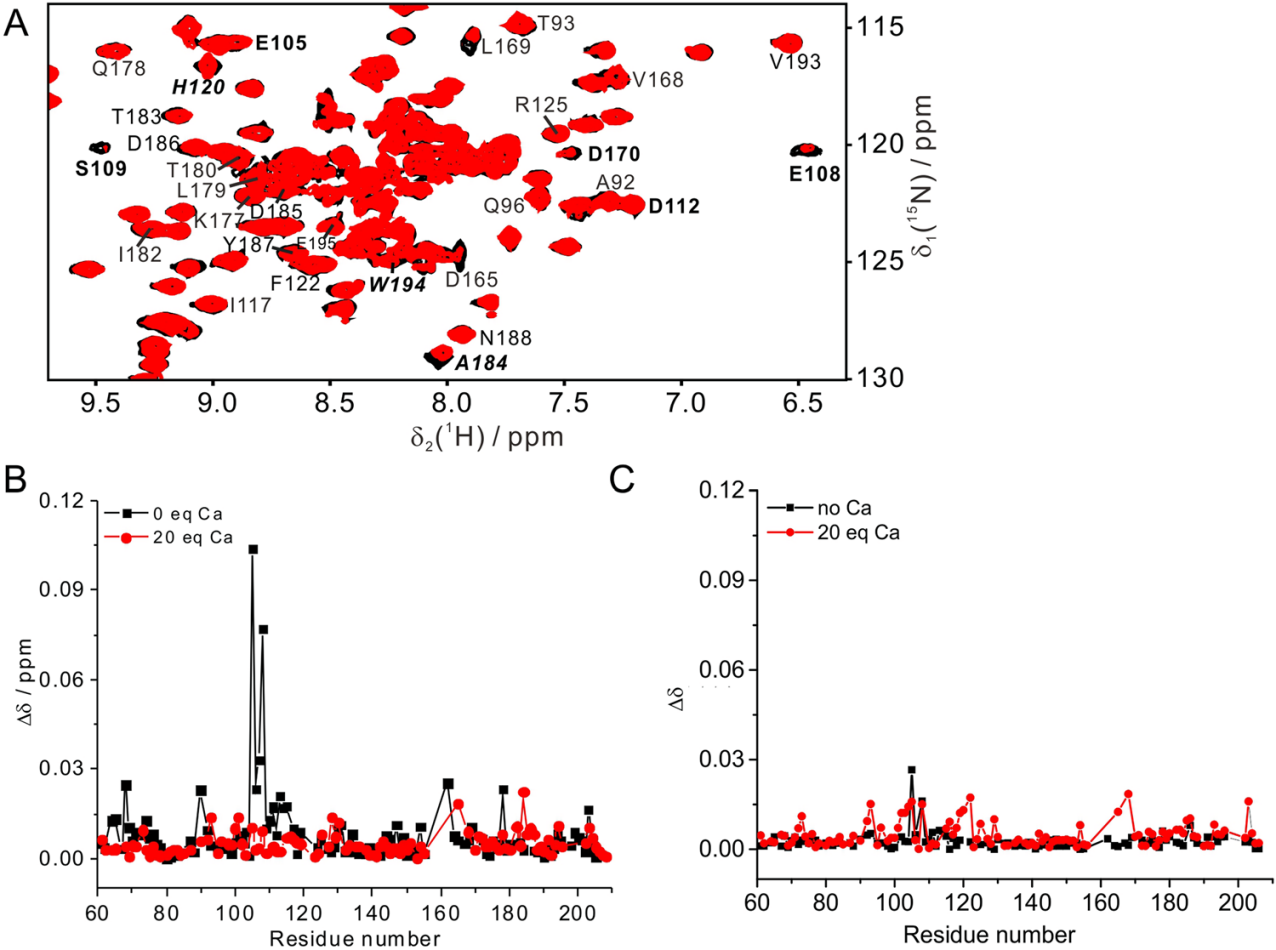
The noncovalent association of SrtA with substrate is weak regardless of the presence or absence of calcium. (**A**) Superimposition of ^15^N-HSQC spectra of 0.1 mM SrtA C184A with 2.0 mM Ca^2+^ without (black) or with 2 mM Ac-LPETG-NH_2_ (red) in 20 mM MES, pH 6.4. Cross-peaks of residues near the calcium binding site are labeled in bold. Cross-peaks of residues near the active site are labeled in bold and italics. (**B**) Chemical shift changes of 0.1 mM SrtA C184A caused by the addition of 2 mM Ac-LPETG-NH_2_ peptide in the presence (red) or absence (black) of 2 mM Ca^2+^ plotted versus the amino acid sequence. (**C**) Same as (**B**) but for the peptide QALPETG-NH_2_.

In the absence of bound calcium, titration of SrtA C184A with substrate peptide produced significant changes in chemical shifts for residues near the calcium binding site, but not near the active site (Fig. 3). More pronounced CSPs were observed with Ac-LPETG-NH_2_ than QALPETG-NH_2_ (Figs 3B and 4B,C), in agreement with the greater reactivity of Ac-LPETG-NH_2_ with wild-type SrtA (Fig. 2). These results indicate that recognition of peptide substrate by SrtA involves the calcium binding motif. The presence of calcium did not alter the magnitude in chemical shift changes, except that the amides of Glu105 and Glu108, which participate in the calcium binding site, became insensitive to the presence of substrate (Fig. 4). Starting with an increased concentration of the protein-Ca^2+^ complex to promote formation of the protein-substrate complex, titration with QALPETG-NH_2_ generated linear chemical shift changes only, indicating that the protein was far from saturation with peptide (Fig. S6A,B). Therefore, the *K*_d_ value of the calcium-bound SrtA C184A–peptide complex must be larger than about 50 mM (Fig. S6C). This indicates that substrate peptides associate with SrtA only weakly, irrespective of the presence or absence of calcium.

### The thioester intermediate binds calcium more tightly than SrtA

**Figure 5:**
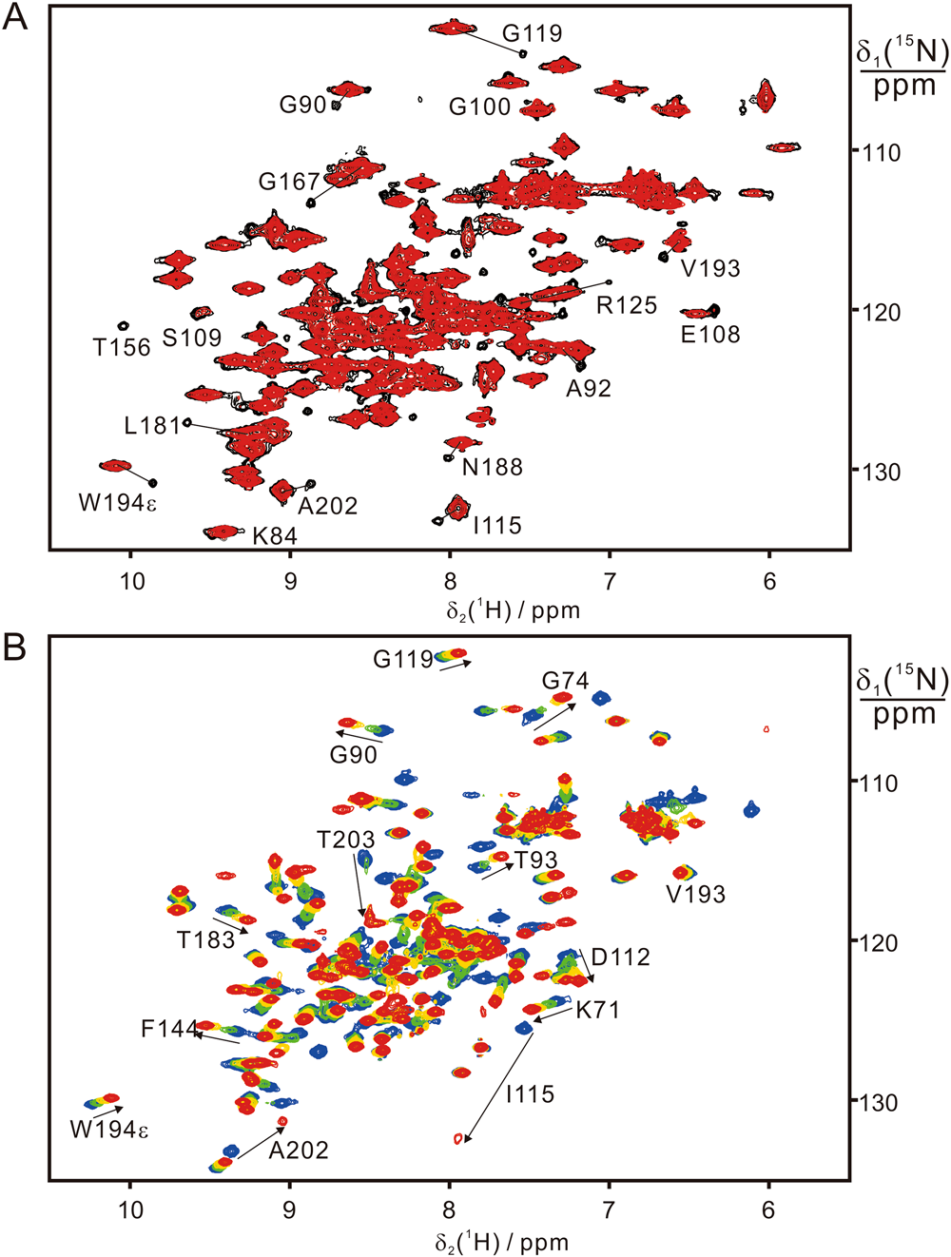
The exchange of SrtA-bound calcium with free calcium is slow on the NMR time scale (μs – ms) in the SrtA-substrate complex and fast in the absence of substrate. (**A**) Superimposition of ^15^N-HSQC spectra of 0.5 mM SrtA and 1 mM Ca^2+^ in the absence (red) and presence (black) of 1 mM QALPETG-NH_2_ (the spectrum was recorded right after addition of peptide because of fast hydrolysis of the unstable thioester in solution) in 20 mM MES, pH 6.4. Slow exchange of calcium is also evidenced by conservation of the chemical shifts of the complex at lower concentrations (see Fig. 6A). (**B**) Titration of 0.15 mM SrtA with 0.0 (blue), 0.5 (green), 3 (yellow), and 10 (red) equivalents of calcium. The gradual change in chemical shifts indicates fast exchange.

**Figure 6:**
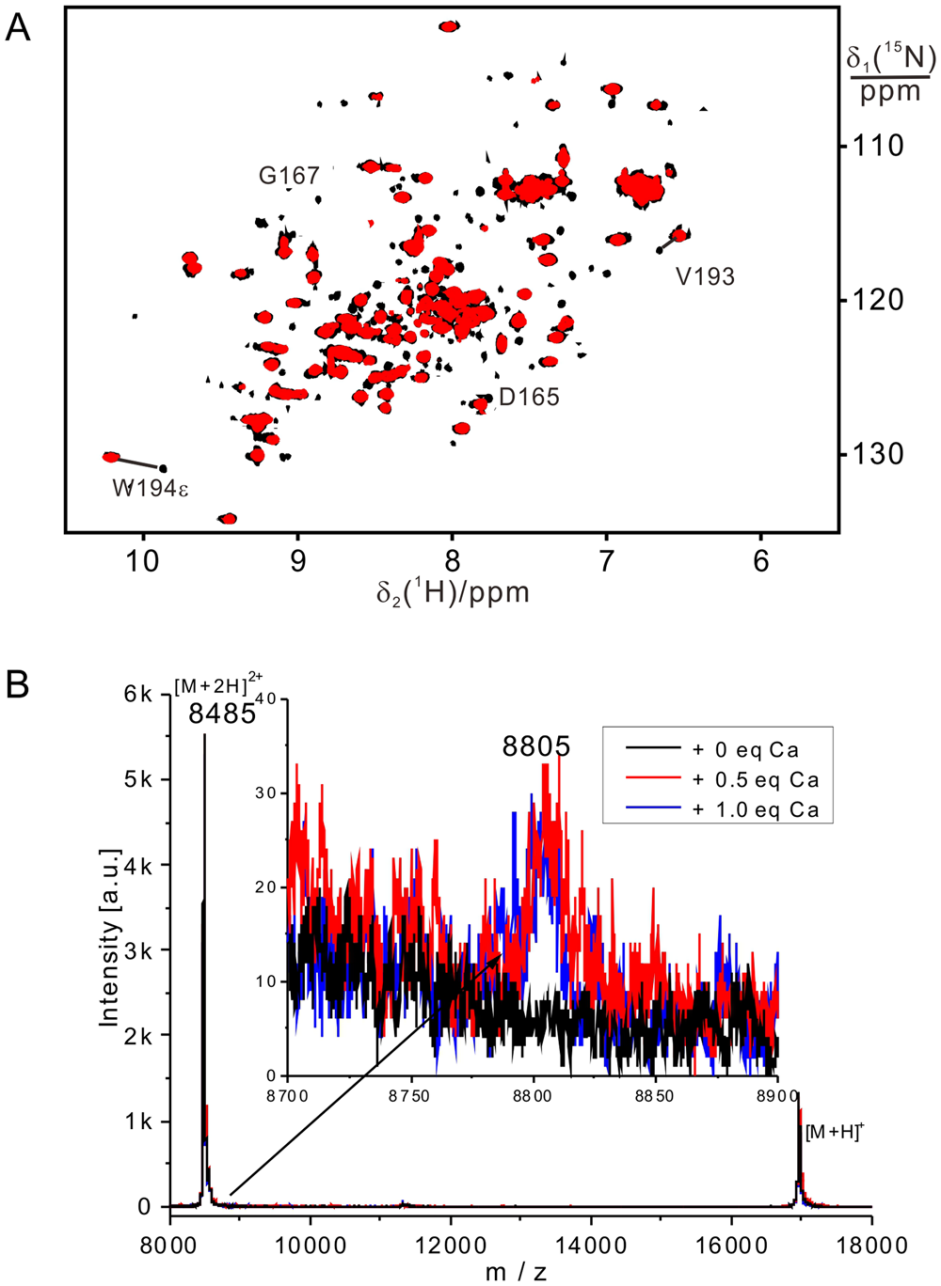
Ca^2+^ ions stabilize the SrtA thioester intermediate at micromolar protein and calcium concentrations. (**A**) Superimposition of ^15^N-HSQC spectra of 50 μM SrtA and 25 μM Ca^2+^ in the absence (red) and presence (black) of 0.5 mM QALPETG-NH_2_ (spectrum was recorded right after addition of peptide) in 20 mM MES, pH 6.4. (**B**) MALDI-TOF mass spectra recorded of a mixture of 10 μM SrtA (uniformly ^15^N-labeled) and 0.1 mM QALPETG-NH_2_ peptide with different concentration of calcium. Black: without Ca^2+^; red: with 5 μM Ca^2+^; blue: with 10 μM Ca^2+^. The inset shows a magnification of the region around m/z = 8800. The difference of 320 mass units observed between SrtA and the thioester with QALPET (m/z = 8485 and 8805, respectively) corresponds to the difference expected for double-charged species.

We next analyzed the binding of calcium to the thioester complex in solution. The thioester (produced in a mixture of 0.1 mM SrtA, 1 mM peptide and 1 mM calcium) hydrolyzes easily and is not stable for long enough to record 3D NMR or 2D NOESY spectra^21^. Therefore we resorted to 2D ^15^N-HSQC spectra to evaluate the binding of calcium. The chemical shifts of the thioester complex with calcium did not vary with calcium concentration (Figs 5A and 6A), indicating slow exchange between bound and free calcium. In contrast, free SrtA binds calcium in fast exchange (Fig. 5B). Remarkably, the calcium complex of the thioester intermediate could be detected at Ca^2+^ concentrations as low as 25 μM by NMR (Fig. 6A) and 5 μM by MALDI-TOF experiments (Fig. 6B), showing that the thioester intermediate binds calcium much more tightly than free SrtA. Removal of calcium by EDTA resulted in quick hydrolysis of the thioester intermediate as evidenced by ^15^N-HSQC and MALDI-TOF experiments (Figs S7 and S8), demonstrating the important role of calcium for stabilizing the unstable thioester intermediate.

**Figure 7:**
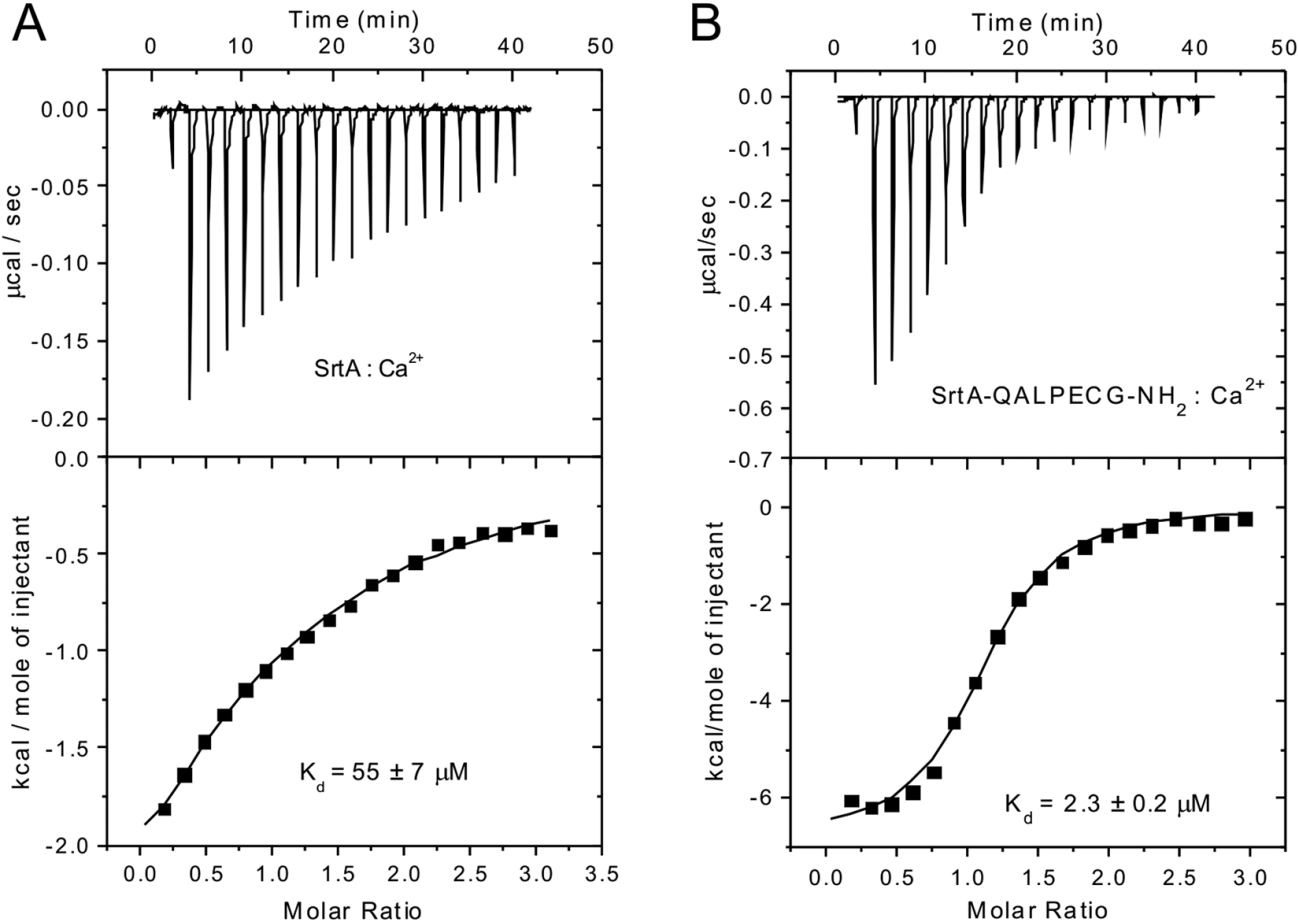
Thioester intermediate complex of SrtA-substrate binds calcium more tightly than free SrtA. ITC measurements of the titration of SrtA (**A**) and the thioester analogue SrtA-QALPECG-NH_2_ (**B**) with CaCl_2_ in 20 mM MES, pH 6.4. The dissociation constants are shown in the figure.

As the thioester intermediate hydrolyzes quickly in the absence of calcium (Figs S7 and S8), we used the previously published thioester analogue SrtA-QALPECG-NH_2_^21^ to quantify its binding affinity with calcium. The thioester analogue contains a disulfide bond between Cys184 of SrtA and the cysteine residue in the QALPECG-NH_2_ peptide, and thus does not hydrolyze^21,22^. Isothermal titration calorimetry (ITC) gave dissociation constants of 2.3 ± 0.3 μM and 55 ± 7μM for the disulfide-linked SrtA-QALPECG-NH_2_ construct and SrtA, respectively (Fig. 7). The greatly enhanced calcium binding affinity of the thioester analogue fully agrees with the experimental data of Fig. 6.

We conclude that the enhanced calcium binding affinity in the thioester intermediate arises from a protein-mediated allosteric effect that depends more on thioester bond formation than on non-covalent interactions with the substrate. Stability of the thioester bond requires the presence of calcium and tight binding of calcium requires the presence of the thioester bond.

### Energy source for thioester bond formation in SrtA catalysis

Thioesters are thermodynamically much less stable than amide bonds, and hydrolysis of the amide bond between the threonine and glycine residue of the LPXTG motif of the substrate alone does not provide sufficient energy to form a thioester bond between the threonine carbonyl group and Cys184 of the enzyme. The shortfall in free energy can be estimated from the free energy of hydrolysis of a C-terminal glycine residue from poly-glycine (−10 kJ/mol) and the free energy of hydrolysis of a thioester (about −18 kJ/mol for the hydrolysis of, e.g., S-acetyl-mercaptopropanol)^26,27^. This energy gap could be bridged by energetically favorable interactions between substrate and enzyme, but the present study shows that *Staphylococcus aureus* SrtA does not form strong non-covalent enzyme–substrate interactions. Instead, our experiments indicate that the missing free energy is provided by the binding of calcium. The free energy ΔΔ*G* delivered by calcium binding can be estimated from the difference in dissociation constants of calcium from the thioester analogue SrtA-QALPECG-NH_2_ (*K*_1_) and free SrtA (*K*_2_)

1 $${\rm{\Delta }}{\rm{\Delta }}G=-\,RT\,\mathrm{ln}({K}_{1}/{K}_{2})=-\,7.9\,\mathrm{kJ}/\mathrm{mol}$$

where *R* is the gas constant and *T* the temperature. This suggests that the increase in calcium binding affinity accompanying the formation of the thioester intermediate provides the necessary free energy to allow accumulation of the thioester intermediate in appreciable quantities. At the same time, an allosteric structure change shields the thioester bond kinetically against hydrolysis, so that an oligo-glycine substrate can compete with water to resolve the thioester bond in the next step of the enzymatic cycle.

## Discussion

In some way, the activity of SrtA is reminiscent of cysteine proteases, which proceed via thioester intermediates but do not protect the intermediates against hydrolytic attack. In the case of SrtA, however, resolution of the thioester intermediate by hydrolysis is an undesired side reaction. Catalysis of the hydrolysis reaction may be discouraged by a move of the side chain of His120 away from Cys184 in the thioester intermediate^21,22^. Spontaneous hydrolysis, however, must also be suppressed while at the same time encouraging reaction with the second substrate, which consists of a relatively bulky oligo-glycine peptide. It is difficult to conceive how this could be achieved without a significant conformational change in the enzyme.

As the role of SrtA is to catalyze peptidoglycan formation and the covalent attachment of virulence factors to the extracellular surface of the bacterium^6^, the enzyme must be active on the cell surface, while premature activity in the bacterial cytosol could be detrimental. The required switch in activity is elegantly achieved by regulation by calcium, which occurs in much higher concentrations in eukaryotic host environments, especially in blood, than inside host or bacterial cells^28^. Indeed, compared with the *K*_d_ value of the SrtA-substrate-calcium complex determined in the present work, the concentration of free calcium in the cytosol of *E*. *coli* is lower and highly regulated^29^. It is likely that *S*. *aureus* similarly controls the level of free cytosolic calcium. Thus, *S*. *aureus* SrtA was found to be inactive in the intracellular space^30^.

Notably, not all sortases depend on metal binding for high enzymatic activity^6,31^. Sortase activity can thus also be achieved in a more classical way by using the binding energy provided by the initial interaction between protein and substrate to bridge the gap in free energy between an amide and a thioester bond. By exploiting the relatively large amount of free energy that can be made available by a metal binding event, *S*. *aureus* SrtA can drive the requisite conformational change without having to rely on a large interfacial area with the substrate peptide, which would be required to deliver a similar amount of free energy.

The abundance of thioester intermediates in biosynthetic pathways, exemplified by the key role of the thioester compound acetyl-CoA, and the similarity in energy that can be gained from hydrolysis of ATP or a thioester bond has led to the proposal of a “thioester world” in an early stage in evolution, which initially may have been devoid of ATP^32^. In such a setting, non-ribosomal peptide bond formation unassisted by nucleotide triphosphates, as achieved by SrtA, could arguably have played an important role. Versatility in the choice of amino acids would be much easier to achieve by enzymes that rely on variable metal binding affinity rather than specific peptide recognition to produce the requisite high-energy intermediates.

## Materials and Methods

### Sample preparation

SrtA (SrtA_ΔN59_, comprising residues 60–206) and its C184A mutant were prepared as described below, with protein expression and purification following previously published protocols^17,21^. The requisite genes were cloned into a PET3a vector. Plasmids were transformed into *E*. *coli* BL21 (Rosetta) cells. Protein expression was induced by isopropyl-D-1-thiogalactopyranoside (IPTG). Unlabeled protein was prepared by growing cells in LB medium and ^15^N-labeled protein was prepared by growing cells in M9 medium using an established high cell density protocol^33,34^. Cells were harvested by centrifugation and lysed by ultrasonication following resuspension in 20 mM Tris-HCl buffer (pH 7.6). Lysate supernatants were collected and the proteins were purified by anion exchange chromatography using an ÄKTA FPLC (GE Healthcare). Pure protein was obtained by Superdex75 gel filtration. Approximately 50 mg purified unlabeled protein was obtained from 1 L LB medium and 20 mg uniformly ^15^N-labeled protein from 250 mL M9 medium.

Substrate peptides were purchased from KE Biochem Co. Ltd (China) and prepared as 30 mM stock in Milli-Q water. The thioester analogue SrtA-QALPECG-NH_2_ was prepared as reported previously (Scheme S1)^21^.

### Enzyme activity measurements

Changes in substrate peptide concentration as a function of incubation time with SrtA were monitored by comparing peak intensities of well-resolved resonances in 1D ^1^H NMR spectra in 20 mM Tris-HCl, pH 7.2, at 298 K. The C-terminal NH_2_ group of Ac-LPETG-NH_2_ and QALPETG-NH_2_ displayed well-resolved NMR signals, which were monitored in the transpeptidation and hydrolysis reactions. The transpeptidation reaction is fast compared with the formation of the thioester intermediate, which accumulates only in the absence of GGG peptide^35^. This allows the loss of substrate peptides to be described as a pseudo-first-order reaction, where the reaction rate constant of transpeptidation was obtained by fitting the incubation time dependent concentration of substrate peptide by the following equation.

2 $${[{\rm{S}}]}_{{\rm{t}}}={[{\rm{S}}]}_{{\rm{eq}}}+({[{\rm{S}}]}_{0}-{[{\rm{S}}]}_{{\rm{eq}}})\exp (-{k}^{{\rm{obs}}}t)$$

where [S]_t_ is the concentration of residual substrate peptide at time *t*, [S]_eq_ the concentration of substrate as the reaction reaches equilibrium, [S]_0_ the initial concentration of substrate peptide, and *k*^obs^ the overall rate constant of the transpeptidation reaction.

### NMR spectroscopy

All NMR spectra were recorded at 298 K, using a Bruker Avance 600 MHz spectrometer equipped with a QCI-cryoprobe. Unless mentioned otherwise, samples were in 20 mM MES buffer, pH 6.4, containing 7% D_2_O (v/v). A 3D NOESY-^15^N-HSQC spectrum with a mixing time of 100 ms was recorded for resonance assignments of SrtA and SrtA C184A. 2D ^15^N-HSQC spectra were generally recorded for a protein concentration of 0.1 mM. ^15^N-HSQC spectra of the unstable thioester intermediate were recorded right after addition of substrate peptide into the solution of protein and calcium. 0.7 mM ^15^N-labeled protein samples were used to record 3D NOESY-^15^N-HSQC spectra. NMR samples for the analysis of transpeptidation and hydrolysis experiments were prepared with 0.5 mM substrate peptides, 0.01 mM unlabeled protein, 0.1 mM CaCl_2_ (or 0 mM Ca^2+^ and 0.2 mM EDTA), 1.0 mM GGG (only for transpeptidation reactions) and 7% D_2_O (v/v) in 20 mM Tris-HCl buffer, pH 7.2. NMR samples for studying the binding between SrtA variants and substrate peptides were prepared with 0.1 mM ^15^N-labeled proteins (unless noted otherwise), 2 mM CaCl_2_ (replaced by 0.5 mM EDTA for calcium-free samples). Titration experiments were conducted by adding substrate peptide to final concentrations of 0.1 mM, 0.5 mM, 1.0 mM, 1.5 mM, and 2.0 mM. NMR measurements of the calcium binding affinity of SrtA variants were performed with 0.1 mM ^15^N-labeled protein, and CaCl_2_ was added gradually from 10 mM stock.

### Isothermal titration calorimetry (ITC)

ITC measurements of the disulfide-linked thioester analogue of SrtA were performed by titration of 500 μM Ca^2+^ into 50 μM SrtA-QALPECG-NH_2_ complex in 20 mM MES buffer, pH 6.4, at 298 K. The experiments were performed in triplicate.

## Electronic supplementary material

Supporting information
