# Supplementary material for: Conversion of an amide to a high-energy thioester by Staphylococcus aureus sortase A is powered by variable binding affinity for calcium
Source: Sci Rep. 2018 Nov 6;8:16371. doi: 10.1038/s41598-018-34752-6 (PMC6219580; doi:10.1038/s41598-018-34752-6)
Supplement: Supplementary file 1 — Supporting information [file 41598_2018_34752_MOESM1_ESM.pdf]

Supporting information

Conversion of an amide to a high-energy thioester by *Staphylococcus aureus* sortase A is powered by variable binding affinity for calcium

Xiao Wang[1], Jia-Liang Chen[1], Gottfried Otting[2], and Xun-Cheng Su*[1]

[1]State Key Laboratory of Elemento-Organic Chemistry, Department of Chemical Biology, College of Chemistry and Collaborative Innovation Center of Chemical Science and Engineering (Tianjin), Nankai University, Tianjin 300071, China

[2]Research School of Chemistry, Australian National University, Canberra, ACT 2601, Australia

Preparation of the disulfide linked thioester intermediate analogue

The thioester analogue containing a disulfide bond between SrtA C184 and the cysteine residue in the QALPECG-NH_2 was prepared as shown in Scheme S1. The disulfide-linked SrtA-QALPECG-NH_2 adduct was purified by a pD10 column. The overall ligation yield was about 85%.

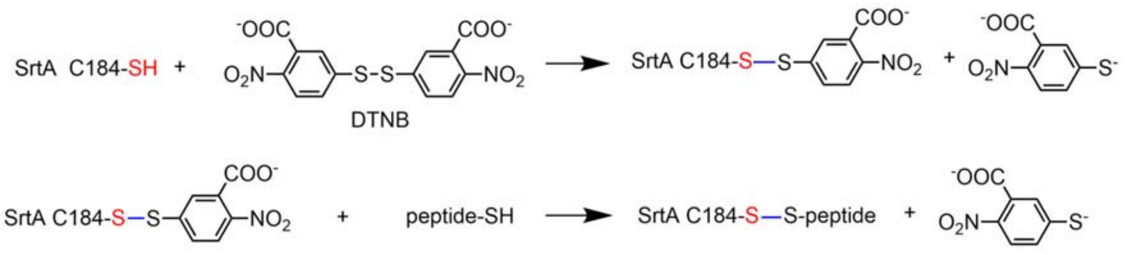

Scheme S1. Ligation reaction producing the disulfide bond-linked thioester analogue SrtA–QALPECG-NH_2.

Peptide	amino acid sequence
1	**LPETG**
2	QA**LPETG**
3	**LPETG**EE-*NH₂*
4	*Ac*-**LPETG**-*NH₂*
5	QA**LPETG**-*NH₂*

Figure S1. Peptides containing the canonical LPETG motif used in this study to analyse the interaction with SrtA and catalysis. *NH_2* denotes an amide group at the C-terminus. *Ac* denotes an acetyl group at the N-terminus.

Table S1. Pseudo-first-order reaction rates of transpeptidation, k^{obs}, of peptide substrate catalyzed by SrtA.[a]

	SrtA (k^{obs} / h^{-1})	
	N^CH_1	N^CH_2
Ac-LPETG-NH_2	0.34±0.02	0.34±0.02
QALPETG-NH_2	0.17±0.01	0.15±0.01

[a] The reaction rate was determined by monitoring the decay of peak intensities of the C-terminal NH_2 group, N^CH_1 and N^CH_2, in 1D ^1H NMR spectra. NMR samples contained 0.01 mM unlabeled protein, 0.5 mM substrate peptide, 0.1 mM $CaCl_2$, 1.0 mM GGG, and 7% D_2O (v/v) in 20 mM Tris-HCl buffer, pH 7.2, at 298 K. The corresponding NMR spectra are shown below, where new signals generated by the reaction were marked as star.

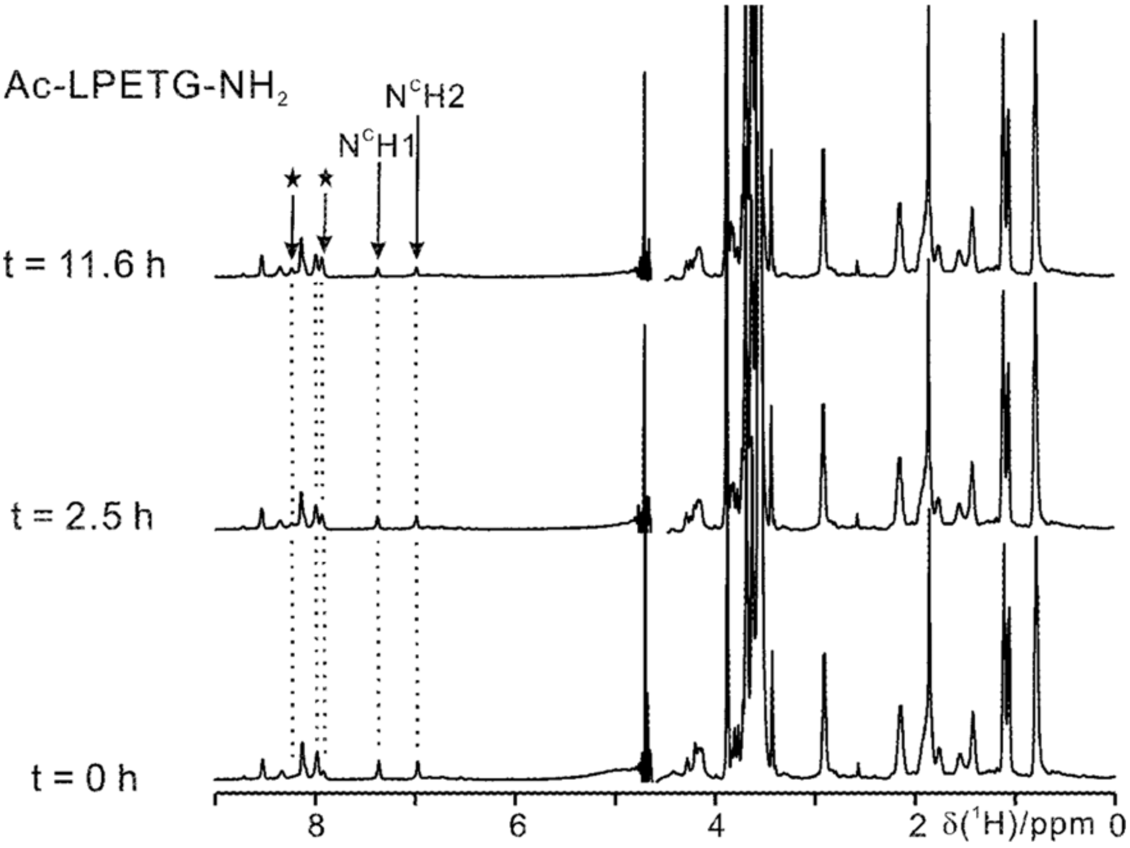

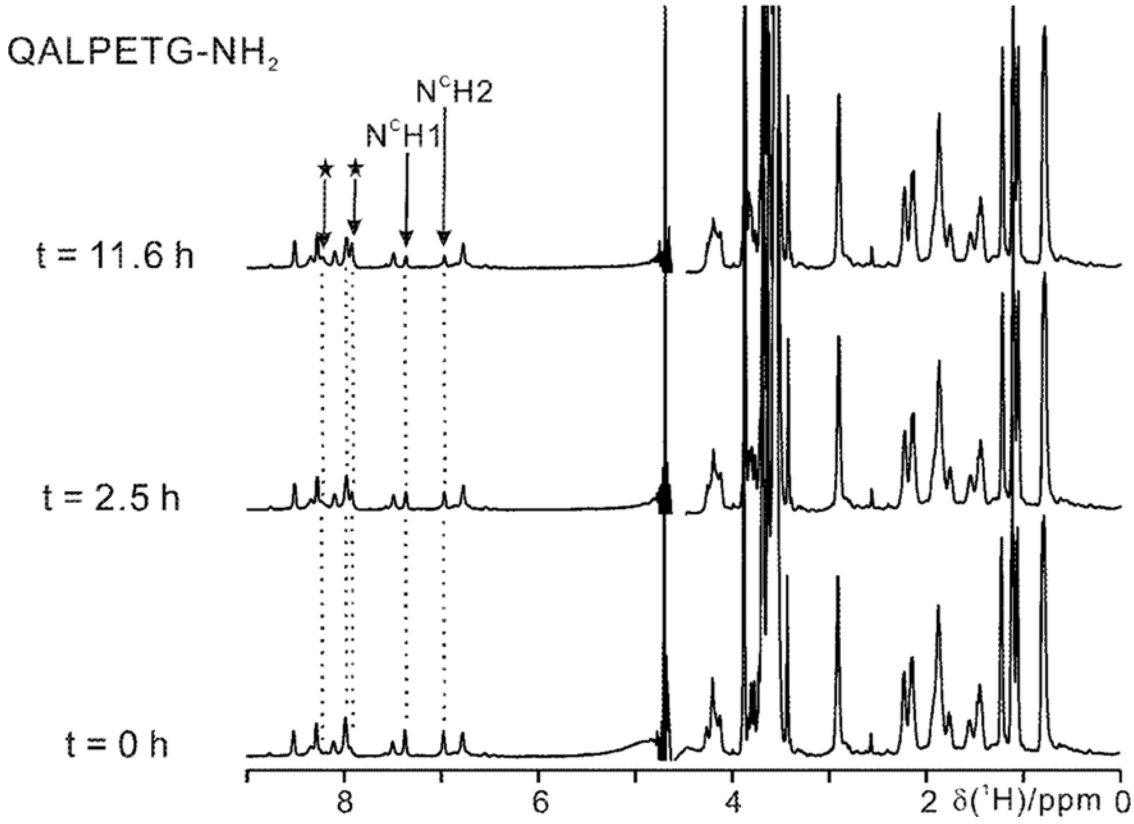

QALPETG-NH$_2$
N^{c}H2
N^{c}H1
★ ★
t = 11.6 h
t = 2.5 h
t = 0 h
8
6
4
2 δ(^{1}H)/ppm 0

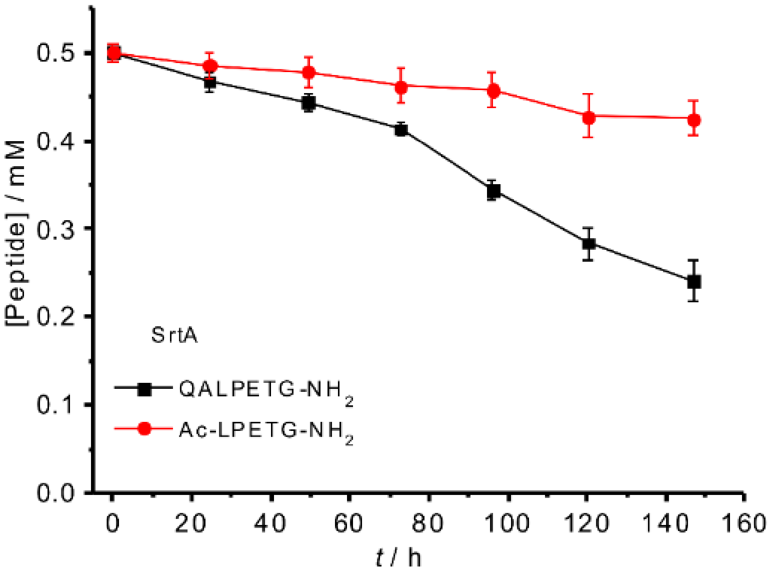

Figure S2. Hydrolysis of substrate peptide catalyzed by SrtA in the absence of GGG peptide. The signals of the C-terminal NH_2 groups of the peptides were monitored by 1D proton NMR for the reaction mixture of 0.5 mM substrate, 0.1 mM Ca^{2+}, and 0.01 mM SrtA in 20 mM Tris-HCl, pH 7.2. Some of the corresponding NMR spectra are shown below, where new NMR signals generated in the reaction were marked as star.

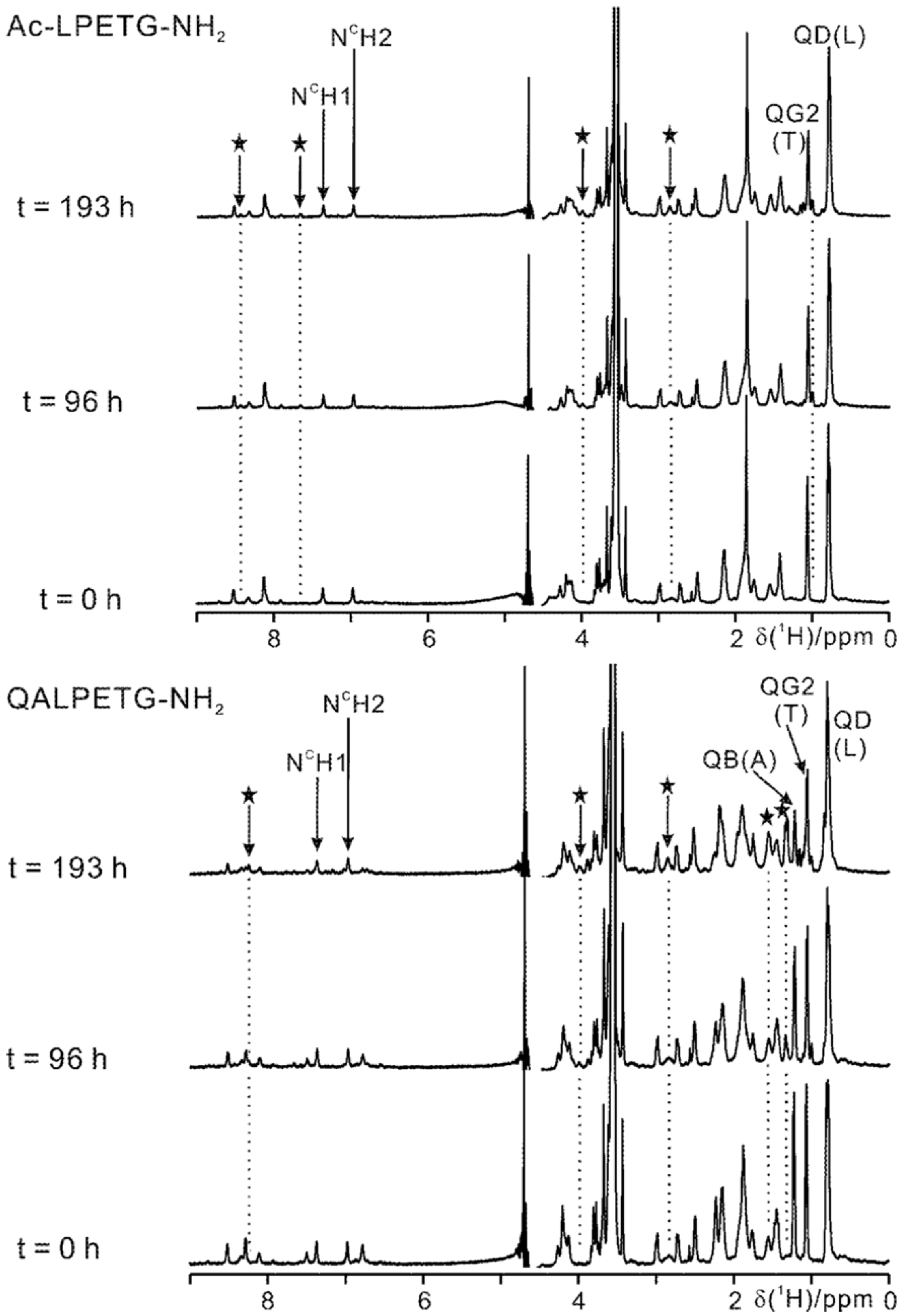

Ac-LPETG-NH$_2$
N^{c}H2
N^{c}H1
QD(L)
QG2 (T)
t = 193 h
t = 96 h
t = 0 h
8
6
4
2 δ(^{1}H)/ppm 0
QALPETG-NH$_2$
N^{c}H2
N^{c}H1
QG2 (T)
QD (L)
QB(A)
t = 193 h
t = 96 h
t = 0 h
8
6
4
2 δ(^{1}H)/ppm 0

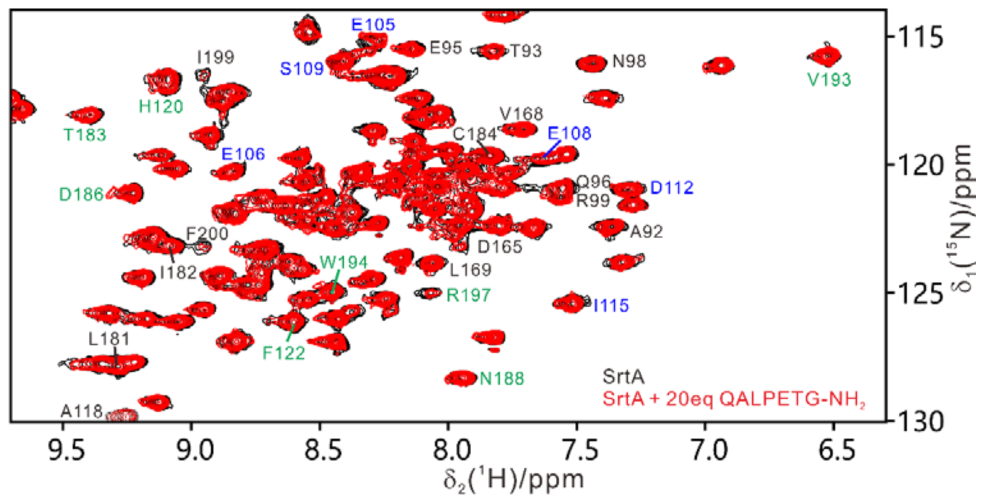

Figure S3. Substrate binding to SrtA is weak in the absence of calcium. Superimposition of ^{15}N-HSQC spectra recorded for 0.1 mM SrtA in the absence (black) and presence (red) of 2.0 mM QALPETG-NH_2. Cross-peaks of residues of the calcium binding site are labeled in blue, those near the active site are labeled in green. Other cross-peaks are labeled in black.

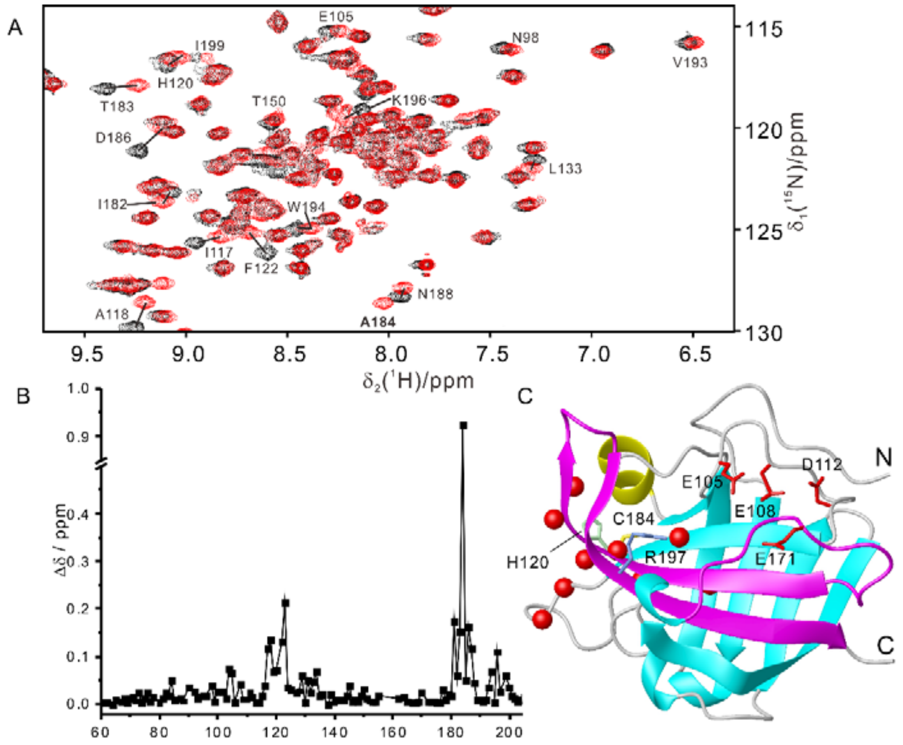

Figure S4. Mutation of Cys184 in SrtA to alanine conserves the protein structure, causing only a local perturbation in chemical shifts. (A) Superimposition of ^{15}N-HSQC spectra of 0.10 mM solutions of SrtA (black) and SrtA C184A (red). Cross-peaks with significantly altered chemical shifts are labeled with their assignment. (B) Chemical shift differences between SrtA and SrtA C184A. In this figure and all following figures, chemical shift perturbations (CSP) were calculated as $\Delta\delta = \mathrm{Sqrt}[(\Delta\delta_H)^2 + (\Delta\delta_N/10)^2]$, where $\Delta\delta_H$ and $\Delta\delta_N$ are the chemical shift differences of backbone amides in the hydrogen and nitrogen dimension, respectively. (C) Structural representation of SrtA (PDB code: 1T2P[1]) highlighting the C^{α} atoms of residues with amide CSPs $\geqslant$ 0.10 ppm in (B) as red balls. The sidechains of E105, E108, D112, and E171 of the calcium binding motif are shown in red, and the sidechains of C184, R197, and H120 in the active site are shown in yellow, blue, and green, respectively.

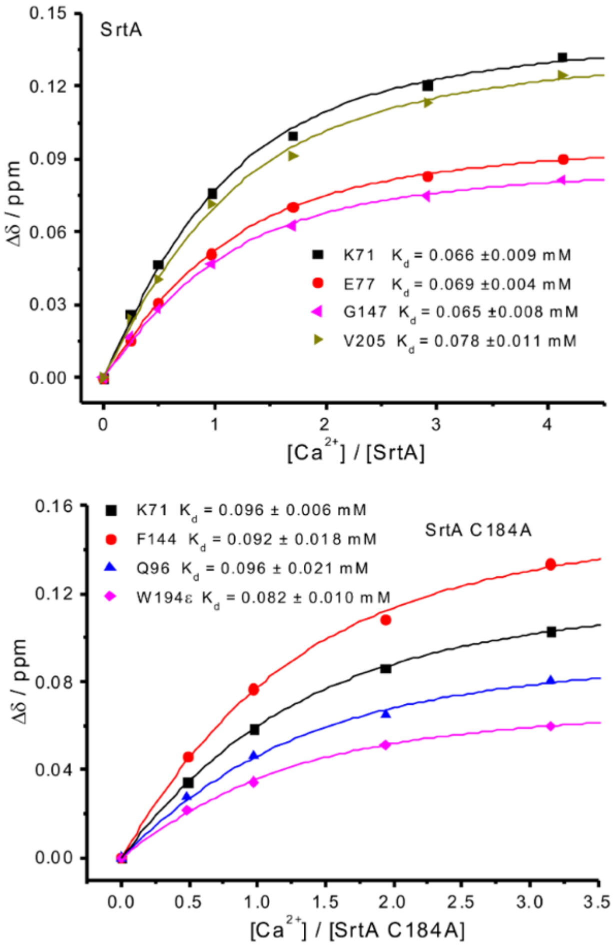

Figure S5. Measurement of the dissociation constant K_d of the calcium complexes of SrtA and SrtA C184A. The K_d values were determined by monitoring the chemical shift changes $\Delta\delta$ of backbone amide protons in ^{15}N-HSQC spectra in titration experiments with 0.1 mM protein as a function of increasing concentrations of calcium. Non-linear curve fitting was performed using the equation $\Delta\delta = 0.5*\Delta*((K/P+x+1)-((K/P+x+1)^2-4*x)^{0.5})$, where Δ is the chemical shift difference between the 1:1 protein-Ca^{2+} complex and the free protein, K the dissociation constant, P the protein concentration, and x the molar ratio of Ca^{2+} to protein.

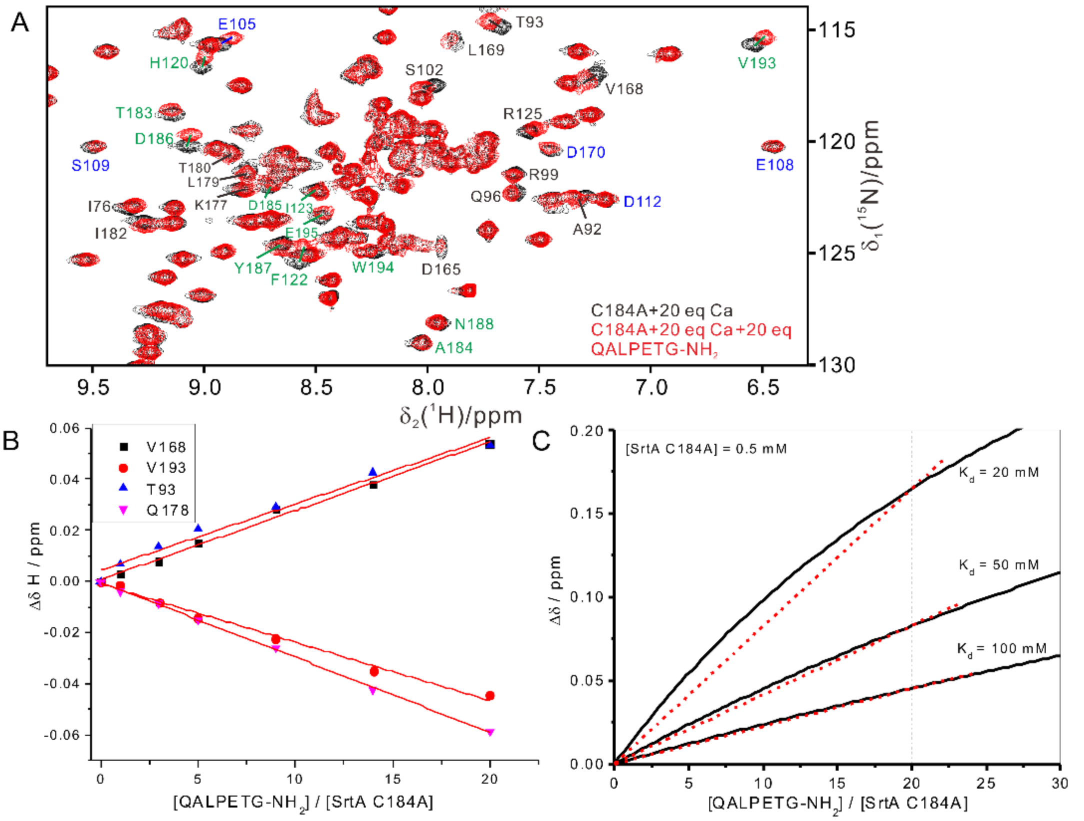

Figure S6. Determining a lower limit for the dissociation constant of the complex between SrtA C184A and QALPETG-NH_2. (A) Superimposition of ^{15}N-HSQC spectra of 0.5 mM SrtA C184A and 10 mM Ca^{2+} in the absence (black) and presence (red) of 10 mM QALPETG-NH_2. At these relatively high concentrations, small chemical shift changes due to peptide binding become observable. (B) Chemical shift changes of backbone amide protons of SrtA C184A with increasing concentrations of QALPETG-NH_2. Solution conditions as in (A). (C) Binding curves (solid black lines) calculated with different K_d values in the range of 0 to 20-fold excess of peptide over SrtA C184A. Black lines assume a maximal change in chemical shift of 0.5 ppm for a 0.5 mM solution of SrtA C184A. Reasonable fits to straight lines (dashed red) in the range of 0 to 20-fold excess of peptide over protein can be obtained for K_d values greater than 50 mM. Curves were fitted using the same equation as in Figure S5.

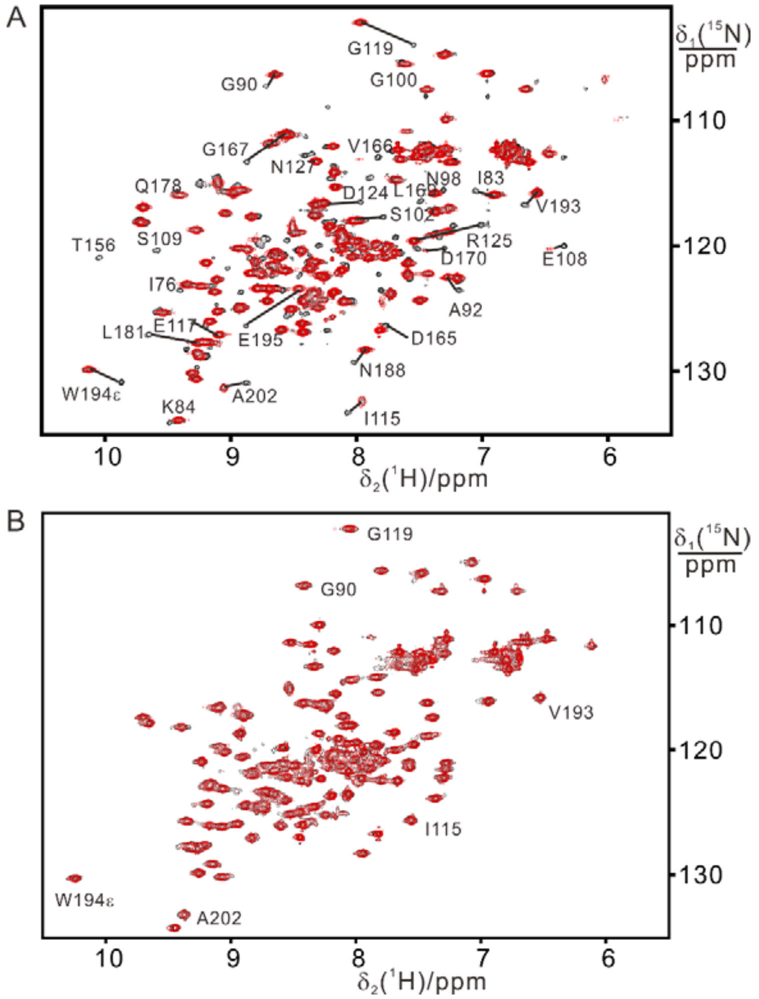

Figure S7. The thioester intermediate hydrolyzes quickly following removal of calcium with EDTA. (A) Superimposition of ^{15}N-HSQC spectra of 0.1 mM SrtA with 1 mM Ca^{2+} in the absence (red) and presence (black) of 1 mM QALPETG-NH_2. The spectrum with peptide was recorded immediately after addition of peptide. (B) Overlay of ^{15}N-HSQC spectra of 0.1 mM SrtA with 1 mM Ca^{2+} (red), and of 0.1 mM SrtA with 1 mM Ca^{2+} incubated with 1 mM QALPETG-NH_2 for 0.5 h followed by the addition of 1.2 mM EDTA (black). Removal of calcium eliminates the cross-peaks from the thioester.

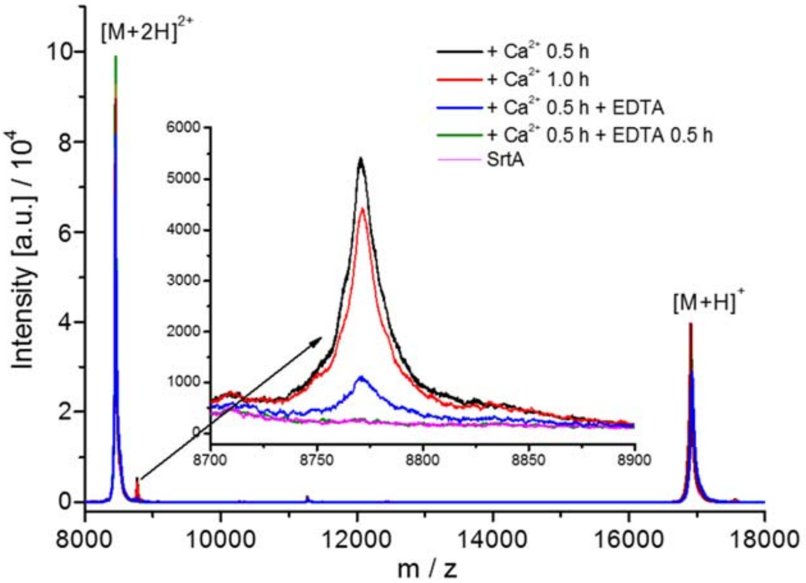

Figure S8. Stability of thioester complex with respect to calcium binding as determined by MALDI-TOF mass spectrometry. A reaction mixture containing 0.1 mM SrtA (in contrast to Figure 5, not isotopically labeled) and 1 mM QALPETG-NH_2 was incubated at 298 K for 0.5 h in the absence of Ca^{2+} (magenta) or for 0.5 h in the presence of 0.5 mM Ca^{2+} (black). The inset shows a magnification of the region around m/z = 8800. The signal of the complex decreased slightly after 1 h in the presence of 0.5 mM Ca^{2+} (red) and substantially after the addition of 5 mM EDTA (blue). No signal of the complex could be observed after incubation of the reaction mixture with 0.1 mM Ca^{2+} for 0.5 h followed by the addition of 5 mM EDTA and further incubation for 0.5 h (green).

References

1. Y. Zong, T. W. Bice, H. Ton-That, O. Schneewind, S. V. Narayana, *J. Biol. Chem*. **2004**, *279*, 31383–31389.
